# Identification and Genetic Characterization of Viral Pathogens in Ruminant Gestation Abnormalities, Israel, 2015–2019

**DOI:** 10.3390/v13112136

**Published:** 2021-10-22

**Authors:** Natalia Golender, Velizar Bumbarov, Anita Kovtunenko, Dan David, Marisol Guini-Rubinstein, Asaf Sol, Martin Beer, Avi Eldar, Kerstin Wernike

**Affiliations:** 1 Department of Virology, Kimron Veterinary Institute, Bet Dagan 50250, Israel; velizarb@moag.gov.il (V.B.); anitak@moag.gov.il (A.K.); dand@moag.gov.il (D.D.); marisolg@moag.gov.il (M.G.-R.); asafs@moag.gov.il (A.S.); eldar@moag.gov.il (A.E.); 2 Institute of Diagnostic Virology, Friedrich-Loeffler-Institut, 17493 Greifswald-Insel Riems, Germany; Martin.Beer@fli.de (M.B.); Kerstin.Wernike@fli.de (K.W.)

**Keywords:** abortion, malformation, fetal infection, cattle, sheep, goat, bunyavirus, orbivirus, pestivirus

## Abstract

Infectious agents including viruses are important abortifacients and can cause fetal abnormalities in livestock animals. Here, samples that had been collected in Israel from aborted or malformed ruminant fetuses between 2015 and 2019 were investigated for the presence of the following viruses: the reoviruses bluetongue virus (BTV) and epizootic hemorrhagic disease virus (EHDV), the flaviviruses bovine viral diarrhea virus (BVDV) and border disease virus (BDV), the peribunyaviruses Shuni virus (SHUV) and Akabane virus (AKAV), bovine herpesvirus type 1 (BoHV-1) and bovine ephemeral fever virus (BEFV). Domestic (cattle, sheep, goat) and wild/zoo ruminants were included in the study. The presence of viral nucleic acid or antigen could be confirmed in 21.8 % of abnormal pregnancies (213 out of 976 investigated cases), with peribunyaviruses, reoviruses and pestiviruses being the most prevalent. At least four different BTV serotypes were involved in abnormal courses of pregnancy in Israel. The subtyping of pestiviruses revealed the presence of two BDV and several distinct BVDV type 1 strains. The peribunyaviruses AKAV and SHUV were identified annually throughout the study period, however, variation in the extent of virus circulation could be observed between the years. In 2018, AKAV even represented the most detected pathogen in cases of small domestic ruminant gestation abnormalities. In conclusion, it was shown that various viruses are involved in abnormal courses of pregnancy in ruminants in Israel.

## 1. Introduction

Embryogenic deaths, abortions and stillbirth in domestic ruminants induce huge economical losses in the farming industry worldwide. The causes of abortion can be numerous and range from infectious agents (bacteria, viruses, protozoa, and fungi) to toxic agents, heat stress, and inherited genetic abnormalities [1,2,3]. Among the infectious agents, viruses of multiple families, such as e.g., *Herpesviridae*, *Flaviviridae*, *Peribunyaviridae* or *Reoviridae*, play a major role in inducing abortion and pregnancy abnormalities [4,5,6,7,8,9,10,11,12]. Within the latter family, the genus *Orbivirus* includes the arthropod-transmitted bluetongue (BTV) and epizootic hemorrhagic disease viruses (EHDV), which are both of veterinary importance, infect cloven-hoofed ungulates and may induce severe and even fatal diseases in susceptible ruminants. Clinical signs caused by BTV and EHDV range from subclinical disease to fever, lameness, coronitis, edema, hemorrhage and ulcers in the oral cavity and upper gastrointestinal tract and necrosis of the skeletal and cardiac muscle [13,14]. Some strains of BTV may cross the placental barrier and induce abortion or lesions in the central nervous system of the developing fetus [13,15].

Israel has been endemic for BTV since the 1940s. During the period of this study (2015–2019), ten different serotypes were identified and isolated (BTV-1, -2, -3, -4, -5, -6, -8, -9, -15 and -16) [16] [Golender et al., unpublished], some of them from aborted ruminant fetuses. In addition, recent EHDV outbreaks revealed that some EHDV field strains found in Israel are probably abortifacient for cattle. The EHDV-6 genome was found in aborted bovine fetuses and placentas at the end of 2015, when a large outbreak of the disease was registered in the country [8].

Another virus group of major veterinary importance is the Simbu serogroup of the family *Peribunyaviridae*, genus *Orthobunyavirus*. Important representatives of the Simbu serogroup are, for example, the Akabane (AKAV), Schmallenberg (SBV) and Shuni (SHUV) viruses, among others. These viruses are also transmitted by insect vectors (mostly by *Culicoides* biting midges), are distributed worldwide, and are known to cause pregnancy abnormalities and severe fetal malformation summarized under the term “arthrogryposis-hydranencephaly syndrome” [11,17]. In adult animals, simbuviruses cause predominantly either none or only mild unspecific clinical signs including fever, diarrhea or decreased milk yield [11,17,18,19]. However, some strains of AKAV or SHUV might occasionally induce encephalitis in adult cattle [18,20,21]. In Israel, outbreaks of AKAV leading to the birth of congenitally malformed calves, lambs and kids were recorded for the first time as early as 1969/1970 [22,23,24,25]. In 2002/2003, further AKAV detections were reported [26,27]. Since 2014, another Simbu serogroup virus, namely SHUV, has been circulating in Israel, causing a massive outbreak of abortions/malformations in domestic ruminants in the year of its first detection [6,28].

Bovine ephemeral fever (BEF), also known as “Three Day Sickness”, is likewise an arthropod-borne viral disease of cattle. It is caused by bovine ephemeral fever virus (BEFV), a member of the genus *Ephemerovirus* in the family *Rhabdoviridae*. In Israel, sporadic infections with BEFV are reported [29], with the last cases registered in 2018–2019 (unpublished).

Pestiviruses are also well-known for inducing abortions and gestation abnormalities in livestock animals. The four classical pestivirus species include bovine viral diarrhea virus type 1 (BVDV-1; syn. *Pestivirus A*), BVDV-2 (*Pestivirus B*), classical swine fever virus (CSFV; *Pestivirus C*), and border disease virus (BDV; *Pestivirus D*). Over the course of the past years, a number of additional, atypical pestiviruses were described in various domestic and wildlife animals [30,31,32,33,34], and some of them are suspected to be involved in gestation abnormalities or diseases of newborns [35,36,37]. Regarding herpesviruses, they are constantly identified in aborted and malformed fetuses in many mammalian species [38,39,40]. In ruminants, bovine herpesvirus type 1 (BoHV-1), an *Alphaherpesvirus*, is a major pathogen which induces a variety of syndromes such as infectious bovine rhinotracheitis (IBR), infectious pustular vulvovaginitis (IPV) in cows, and infectious balanoposthitis (IBP) in bulls [41]. However, despite of being well-known as important pathogens, the situation in Israel regarding herpesviral and pestiviral infections of ruminants is largely unknown.

The aim of the present study was to investigate samples obtained from cases of ruminant abortion and neonatal abnormalities, which were submitted for routine diagnostics, for the presence of diverse abortifacient viruses. The samples were collected between 2015 and 2019. In addition, detected BTV strains and Simbu serogroup viruses are further described, since it became apparent that these viruses represent major causes of abortions or pregnancy abnormalities in Israel.

## 2. Materials and Methods

### 2.1. Field Samples

Samples from a total of 976 aborted or malformed domestic or wild ruminant fetuses submitted between 2015 and 2019 for routine examination to the virology department of the Kimron Veterinary Institute (KVI), Israel, were included in this study. Clinical specimens comprised placenta, brain and internal organs from aborted fetuses, and whole blood, plasma and serum samples from abnormal newborns. Details on the number per year, sample matrix, and animal species from which samples were collected are summarized in Table 1.

### 2.2. Viral RNA/DNA Extraction

Tissue samples were homogenized in phosphate buffered saline (PBS). Viral RNA was extracted from all field samples and virus isolates (tissue culture supernatant; please see below) using the Invisorb Spin Virus RNA Mini Kit (STRATEC Molecular, Berlin, Germany), or by the MagMAX™ CORE Nucleic Acid Purification Kit (Thermo Fisher Scientific), according to the manufacturer’s recommendations. For DNA extraction, the Patho Gene-spin™ DNA/RNA Extraction Kit (iNtRON Biotechnology, South Korea) or the MagMAX™ CORE Nucleic Acid Purification Kit (Thermo Fisher Scientific) were used.

### 2.3. Criteria for Laboratory Testing for Different Viruses and Methods Used

The testing for the following viral pathogens was included in this study: orbiviruses (BTV and EHDV), simbuviruses, BoHV-1, pestiviruses (BVDV and BDV), and BEFV. The decision for molecular investigation for BTV, EHDV, simbuviruses, BoHV-1 or BEFV in 2015, 2016 and 2017 was based on epidemiological, clinical, and pathological data, as well as on the availability of “target organs” for a specific pathogen. The investigation was not performed systematically in 2015–2017, meaning that not every sample of every fetus or malformed newborn was analyzed for every pathogen. Since 2018, most cases were systematically screened for the presence of viral abortifacients.

Due to accumulated data on the detection of the BTV and EHDV genomes by RT-PCR in placenta and brain tissues from aborted ruminant fetuses [8,16], sample material for laboratory investigation of orbiviruses have included, since 2018, placenta, brain, spleen and/or lung samples (if available). Before 2018, aborted material was only investigated for the presence of EHDV when the veterinary practitioners that collected the specimens asked for these analyses, or when the samples were collected as a part of EHD outbreak investigations [8,42]. For EHDV detection, the EHDV Real-Time PCR Kit (pan-EHDV RT-PCR) (Applied Biosystems, Thermo Fisher Scientific Inc., Lissieu, France) was used according to the manufacturer’s instructions. For BTV detection, the VetMAX™ BTV NS3 All Genotypes Kit (Applied Biosystems™, Thermo Fisher Scientific Inc., Lissieu, France) was applied. BTV-positive samples were subsequently tested by in-house gel-based RT-PCRs with type-specific primers and with generic primers that enabled subsequent serotyping by sequencing (Supplementary Table S1). A previously published BTV-9 and BTV-15 specific real-time RT-PCR was used for identification of BTV-9 and BTV-15 [43].

Tests for the detection of Simbu serogroup viruses were done continuously throughout the whole study period. Sample materials included, depending on availability, placenta, brain, internal organs and blood. During January to August 2015, diagnostic tests for simbuviruses were type-specific for SBV, AKAV and SHUV, and were performed according to published protocols [44,45,46]. From September 2015 onwards, a generic real-time RT-PCR was applied for detection of simbuviruses, followed by virus identification by partial sequencing of the S-segment using the same pair of primers as used for detection [28]. Further, the M- and L-segments of some AKAV and SHUV strains were partially sequenced; the primers are given in Supplementary Table S1 and the procedure is described in Section 2.4.

The analyses for the presence of pestiviruses was likewise performed throughout the study period. Usually, pools of mixed samples from internal organs, brain, and, if available, placenta tissues were used. In cases where only the placenta was submitted, this material was used for investigation, even in the absence of fetal organs. From 2015 to 2019, most of the samples were tested by a BDVD Ag ELISA (BVDV Ag Serum/Plus HerdChek, IDEXX Laboratories, Westbrook, USA). In 2018 and the first half of the year 2019, the samples were additionally tested by a panpesti real-time RT-PCR [47], which enables the detection of BVDV and the ovine BDV. In the second half of 2019, only the panpesti real-time RT-PCR was applied. Attempts of virus identification in pestivirus positive samples and further subtyping were done by sequence analyses of the 5′ untranslated region (5′ UTR) as described previously [48] (Supplementary Table S1).

In 2018 and 2019, most of the submitted samples from aborted cattle fetuses were additionally tested for BEFV and BoHV-1 genomes. To detect BEFV, a previously published real-time RT-PCR was used [29]. The presence of BoHV-1 DNA in aborted cattle fetuses/placentas was determined according to the method described in the OIE manual (https://www.oie.int/fileadmin/Home/eng/Health_standards/tahm/3.04.11_IBR_IPV.pdf; pp. 1145–1146; accessed on 19 October 2021).

### 2.4. Sequencing and Phylogenetic Analyses

Primers used for partial sequencing of orbiviruses and simbuviruses are listed in Supplementary Table S1. For all conventional RT-PCRs, the One-Step RT-PCR kit (Qiagen, Hilden, Germany) was used. The cDNA fragments of positive samples were purified with the MEGAquick-spin Total Fragment DNA Purification Kit (iNtRON Biotechnology, Gyeonggi-do, South Korea) and subsequently sequenced by standard Sanger methods in both directions using an ABI 3730xl DNA Analyzer (Hylabs, Rehovot, Israel). The resulting nucleotide sequences were assembled and nucleotide (nt) sequences were aligned and pairwise compared by using Geneious version 9.0.5 (Biomatters, Auckland, New Zealand). Phylogenetic trees were constructed using the Mega X software [49]. For all trees the maximum-likelihood method was used and the Tamura Nei (simbuviruses, orbiviruses) and Kimura 2-parameter (pestiviruses) models were applied, respectively.

For full genome sequencing of the ISR-256/16 AKAV strain, RNA from cell culture supernatant was isolated using the Invisorb Spin Virus RNA Mini Kit (STRATEC Molecular, Berlin, Germany) with DNase I (A&A BIOTECHNOLOGY, Gdynia, Poland) treatment before the second washing step. The library was prepared using the Nugen Trio-RNA Seq kit (https://www.nugen.com/products/trio-rna-seq-library-preparation-kit, accessed on 23 August 2021) at Syntezza Bioscience, NGS lab, Jerusalem, Israel, with rRNA depletion. The sequencing run was performed at the University of Illinois Chicago Genomics Research Core Lab on an Illumina MiniSeq instrument using mid output cartridge. The sequences were assembled using Geneious version 9.0.5 (ISR-256/16). The strain ISR-170/18 was sequenced at the Friedrich-Loeffler Institut, Greifswald–Insel Riems, Germany. RNA of this strain was extracted using Trizol Reagent (LifeTechnologies, Darmstadt, Germany) in combination with the RNeasy Mini Kit (Qiagen, Hilden, Germany). Thereafter, cDNA was synthesized by a combination of the SuperScript IV First-Strand cDNA Synthesis System (Invitrogen, Carlsbad, CA, USA) and the NEBNext Ultra II Non-Directional RNA Second Strand Synthesis Module (New England Biolabs, Ipswich, MA, USA). Ion Torrent-compatible libraries were generated using the GeneRead DNA Library L Core Kit (Qiagen, Hilden, Germany) and IonXpress Barcode Adapter (Life Technologies, Darmstadt, Germany). Using an Ion Torrent S5 XL, libraries were sequenced on an Ion 530 chip in 400-bp mode.

All sequences obtained in this study were uploaded to the INSDC under the accession numbers listed in Table S2.

### 2.5. Virus Isolation

Virus isolation in cell culture was attempted from a few selected samples. The selection was based on quantification cycle (Cq) values (only highly positive samples were chosen). For isolation of simbuviruses, African green monkey kidney cells (Vero) and/or baby hamster kidney cells (BHK-21) were applied as described previously [50]. For isolation of pestiviruses and herpesviruses, Madin-Darby bovine kidney (MDBK) cells were used. For inoculation of cells, tissue homogenates were centrifuged and the supernatant was filtrated trough 0.22 µm filter. Sub-confluent cell monolayers were inoculated and incubated for 2 h at 37 °C. Thereafter, the cells were washed twice with PBS, and Dulbecco’s Modified Eagle Medium (DMEM, Biological Industries, Israel), supplemented with 2% fetal calf serum (Gibco, Thermo Fisher, Waltham, MA, USA) was added. Cells were observed daily for cytopathogenic effect (CPE). Usually, three blind passages were performed. For confirmation of pestivirus growth, BVDV Ag ELISA or panpesti RT-qPCR was applied. Attempts to isolate BTV were done in embryonated chicken eggs according to a standard procedure [8].

## 3. Results

### 3.1. Detection of Viral Genomes or Antigen

The presence of viral nucleic acid or antigen could be confirmed in 21.8% of abnormal pregnancies investigated between 2015 and 2019 (213 out of 976 cases, 95% confidence interval (CI) 19.27 to 24.55%). The detailed data are given in Table 1.

A total of 68 samples from 49 cases of abnormal gestation were tested for the presence of EHDV, and 13 samples from 11 cases were found to be positive (13/68, 19.1%, 95% CI 11.77 to 36.62%). Of those, 13/35 tested positive in 2015, 0/27 in 2016, 0/3 in 2017, and 0/3 in 2019). In four out of 11 abortion cases, EHDV-6 was confirmed by sequencing in a previous study [8].

For BTV detection, a pan-BTV RT-qPCR was used and a total of 612 samples of 439 abortion cases were investigated. Thirty-two samples from 27 animals tested positive (32/612, 5.2%, 95% CI 4.09 to 8.82%). In 2015, three out of seven samples (42.86%) were found to be positive. In 2016, only one out of 35 samples (2.86%) scored positive and in 2017 every sample tested negative (0/18, 0% positive). In 2018, a total of 268 samples was investigated and BTV could be detected in 17 (6.34%), while in 2019, 11 out of 284 samples (3.87%) tested positive. For details on serotyping and phylogenetic analyses of the detected BTV strains, please see Section 3.3.2. (Identification and analyses of bluetongue viruses).

For simbuviruses, 155 samples from 134 cases out of 1111 tested samples from 907 abortion cases were positive (13.95%, 95% CI 11.39 to 16.83%; 15/145 in 2015, 40/264 in 2016, 9/171 in 2017, 67/274 in 2018, and 24/258 in 2019). Both AKAV and SHUV were found in tissue samples collected from abnormal ruminant gestations in Israel (Table 1); for detailed phylogenetic analyses of the detected virus strains please see Section 3.3.1. SBV was not detected in any sample.

For pestiviruses, a total of 697 samples of 697 animals were tested by BVDV Ag ELISA, and 52 samples/abortion cases were found to be positive (7.46%, 95% CI 5.62 to 9.67%; 17/171 [9.88%] in 2015, 16/187 [8.56%] in 2016, 8/155 [5.16%] in 2017, 8/144 in 2018 [5.56%], and 3/40 [7.50%] in 2019). Using the panpesti RT-qPCR, 428 samples of 367 animals were tested and 13 samples from 11 animals tested positive (3.04%, 95% CI 1.63 to 5.14%; 3/231 [1.29%] in 2018, and 10/197 [5.08%] in 2019).

A total of 124 samples of 69 cattle fetuses or newborn animals were investigated for BoHV-1 and BEFV genomes, respectively (32 in 2018 and 92 in 2019). None of the samples tested positive for BEFV, while BoHV-1 DNA was detected in two brain samples (one each in 2018 and 2019), representing a BoHV-1 detection rate of 1.6% (95% CI 0.35 to 10.08%).

In rare cases, co-infections with two viruses were detected: BTV and a pestivirus were found in the same animal in two cases (in one ovine fetus each in 2015 and 2019), EHDV-6 and a simbuvirus were identified in one bovine abortion case, and in another cattle abortion mixed infection with EHDV-6 and a pestivirus was detected in 2015. BTV and AKAV were found in the brain and placenta samples of two ovine abortion cases in 2018. A double infection of SHUV and BTV was registered in 2019 in a bovine placenta. The Simbu serogroup viruses AKAV and SHUV were present at the same time in one bovine and one ovine placenta in 2015.

### 3.2. Virus Isolation

In 2016, AKAV was successfully isolated from brain samples of two aborted ovine fetuses and one newborn lamb in cell culture (strains ISR-254/16, ISR-256/16 and ISR-2497/16). In 2018, another AKAV strain was isolated from an aborted ovine fetus in cell culture (ISR-170/18); the sample was collected in July at the beginning of a large AKAV outbreak.

Besides AKAV, BDV was successfully isolated in cell culture; the corresponding sample was obtained from an aborted ovine fetus in 2019 (strain ISR-221/19).

### 3.3. Sequencing and Phylogenetic Analyses of Viruses Detected in Abnormal Ruminant Gestations

#### 3.3.1. Identification and Analysis of Simbu Serogroup Viruses (AKAV and SHUV)

In 2015, 2017 and 2019, when the overall number of submitted samples was small, AKAV was occasionally detected in samples of malformed ruminant fetuses (Table 1). In 2015, for instance, AKAV was found in two out of 36 (5.56%) investigated ovine brain samples, in 2017 in one out of 43 (2.33%) and in 2019 in four out of 72 (5.56%) tested ovine brain samples (Table 1). In 2016, nine out of 81 (11.11%) investigated ovine brain samples tested positive for the AKAV genome. Similar to 2016, in 2018 an increase in abortion cases was seen and AKAV was frequently found, especially in sheep. Twenty out of 46 ovine placenta samples (43.48%) and 25 out of 136 brain samples (18.38%) tested positive for the AKAV genome.

**Figure 1:**
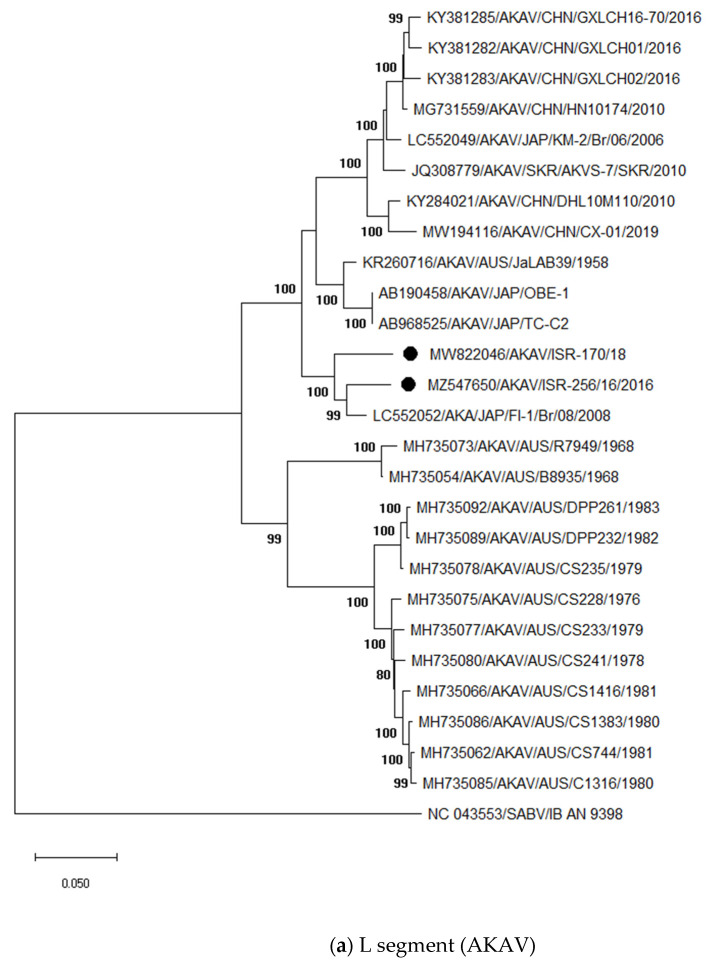

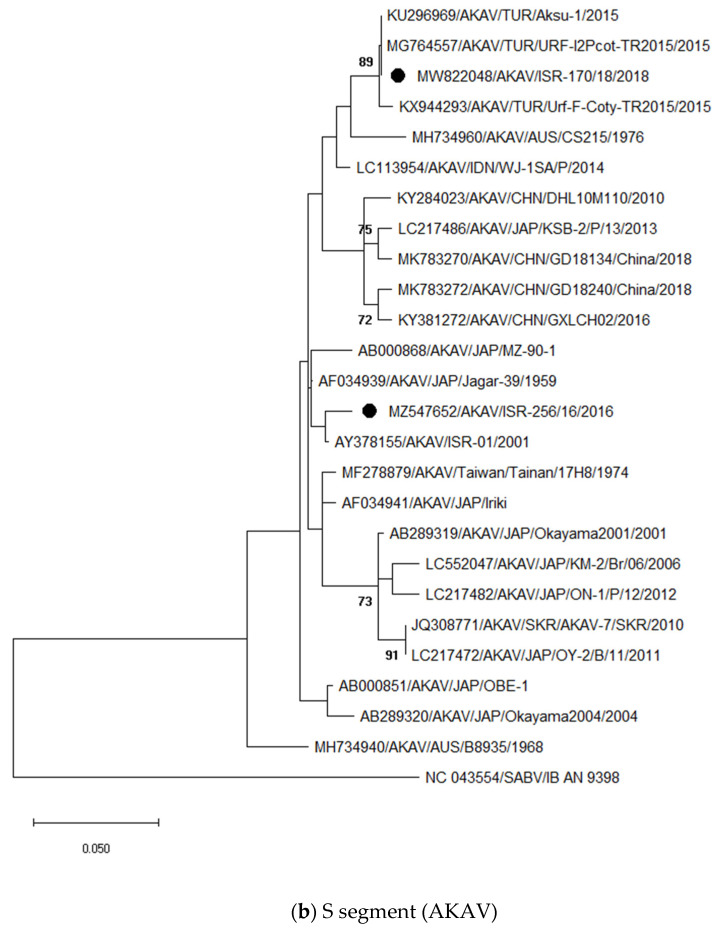

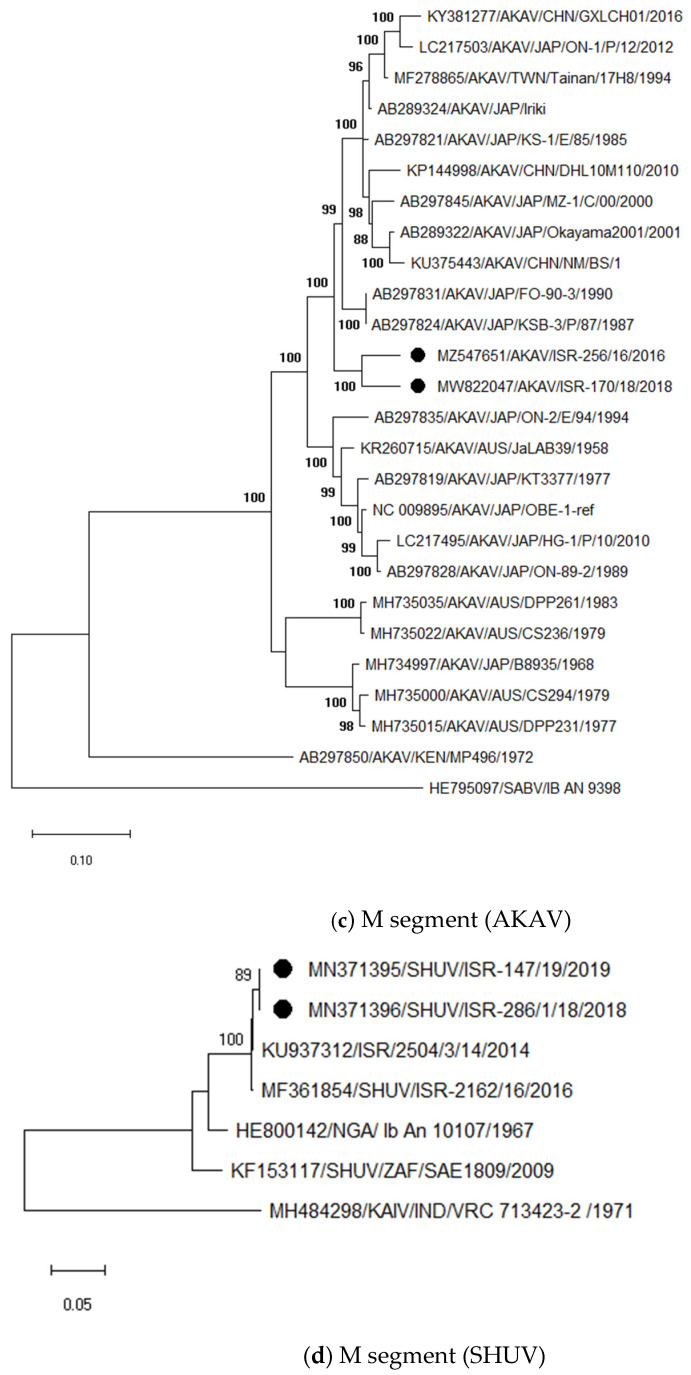
Phylogenetic analyses of the Akabane and Shuni viruses (AKAV and SHUV) based on nucleotide sequences of the L (**a**), S (**b**) and M segment (**c**,**d**). The sequences obtained from NCBI GenBank are labelled by accession number/virus/location/isolate/year (when all data is available in GenBank or the corresponding publications) and sequences generated in the present study are marked by black dots. Nucleotide sequences were analyzed using the Maximum Likelihood method and Tamura-Nei model. Statistical support for nodes was obtained by bootstrapping (1000 replicates); only values ≥ 70 % are shown. Scale bars indicate nucleotide substitutions per site.

For phylogenetic analyses (Figure 1), full-length sequences of all three segments were generated from two AKAV strains (ISR-256/16 and ISR-170/18) and partial sequences from 14 additional strains collected from 2015 to 2018 were also generated (Supplementary Table S2). Phylogenetic analyses of the full-length L segment revealed a close relationship of the Israeli strains ISR-256/16 and ISR-170/18 to the AKAV strain FI-1/Br/08, which was detected in 2008 in Japan (Figure 1a). The nucleotide (nt) identities between strains ISR-256/16 and ISR-170/18 and the Japanese AKAV strain are 94.65% and 96.09%, respectively. Pairwise comparison of the Israeli strains ISR-256/16 and ISR-170/18 revealed a nt identity of 93.63% for the L-segment. The strains for which only partial sequences are available showed the highest identity to strain ISR-256/16 (99.20–100% for strains detected in 2016 and 98.44–99.73% for strains from 2018).

The S segment of the Israeli strain ISR-256/16 showed the highest nt identity (98.43%) to an Israeli AKAV that was isolated from *Culicoides* midges already in 2001 (ISR-01), while strain ISR-170/18 was identical to nearly 100% to several strains identified in Turkey in 2015 from aborted fetuses. Unfortunately, M and L segment sequences are not available for these Turkish strains and, therefore, further analyses and suggestions for the origin of the most recent Israeli strains are not possible.

Comparison of the partial S segment sequences of strains collected in 2015 or 2016 revealed a nt identity of 99.62–99.76% to each other, whereas strains collected in 2018 showed an identity of 99.74–100% to each other.

The M segment sequences of the Israeli AKAV strains cluster closely together (92.52% of nt identity) and to the Japanese strains KSB-6/E/90 and KSB-3/P/87 (Figure 1c). No insertions or deletions were identified in those two strains. However, when comparing M segment sequences, one should keep in mind that sequence mutations accumulate in malformed fetuses in this genomic segment and that these variant viruses do not fully represent circulating strains [51]. Therefore, accumulated mutation in the M segment sequences generated in this study could be higher than in circulating AKAV, i.e., in strains found in viremic adult ruminants or insect vectors. Therefore, the M segment sequences generated in this study should not be used for molecular epidemiology.

Besides AKAV, another Simbu serogroup virus, namely SHUV, was identified by the applied generic pan-Simbu-PCR in samples collected from aborted or malformed ruminants. SHUV was occasionally found in every year covered by this study (Table 1). All identified SHUV strains were closely related to previous Israeli strains (Figure 1d). Partial S segment sequences of 215 bp showed an identity of 100% to each other (these sequences were not submitted to the GenBank). Partial M segment sequences of the strains ISR-147/19 and ISR-286/1/18 showed the closest nt identity to each other (99.74%) followed by strain 2504/3/14 (98.79 and 99.08% of nt identity, respectively). Like for the M segment of AKAV, we cannot definitely appellate with this analysis the origin of the stains, because of the high sequence mutations rates in aborted fetuses.

#### 3.3.2. Identification and Analyses of Bluetongue Viruses

In 2015 and 2016, BTV was detected in a few samples (Table 1); however, serotyping was not performed. In 2017, no BTV was detected. In 2018, the BTV genome was found in 17 samples obtained from 16 animals (15 sheep and one cattle; Table 1). In 2019, 12 samples collected from eight animals were positive (six sheep, one cattle and one goat; Table 1). Type-specific PCRs and sequence analyses of the detected BTV strains revealed that at least four different serotypes have been causing abortion in Israeli domestic ruminants in 2018 and 2019: BTV-3, -4, -9 and -15 (and probably also BTV-8).

**Figure 2:**
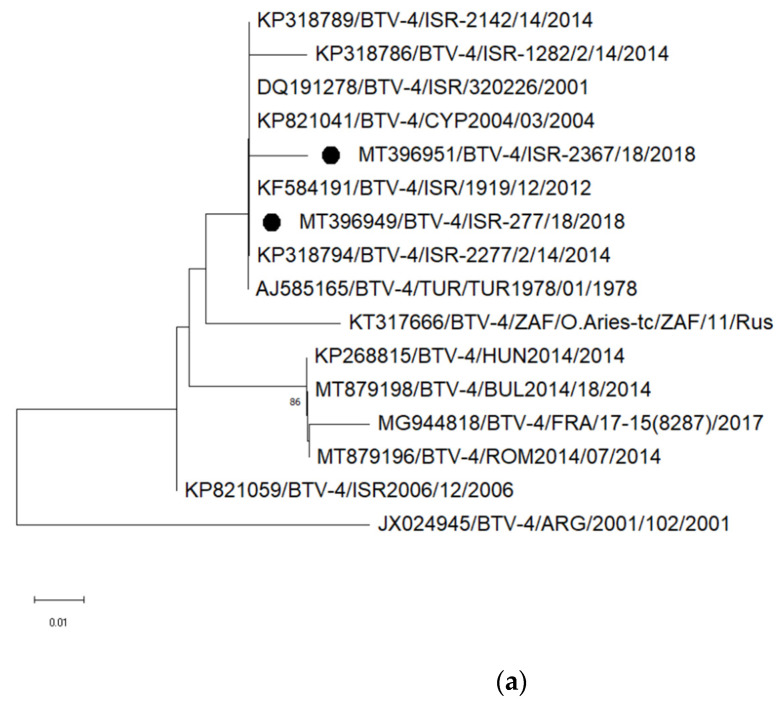

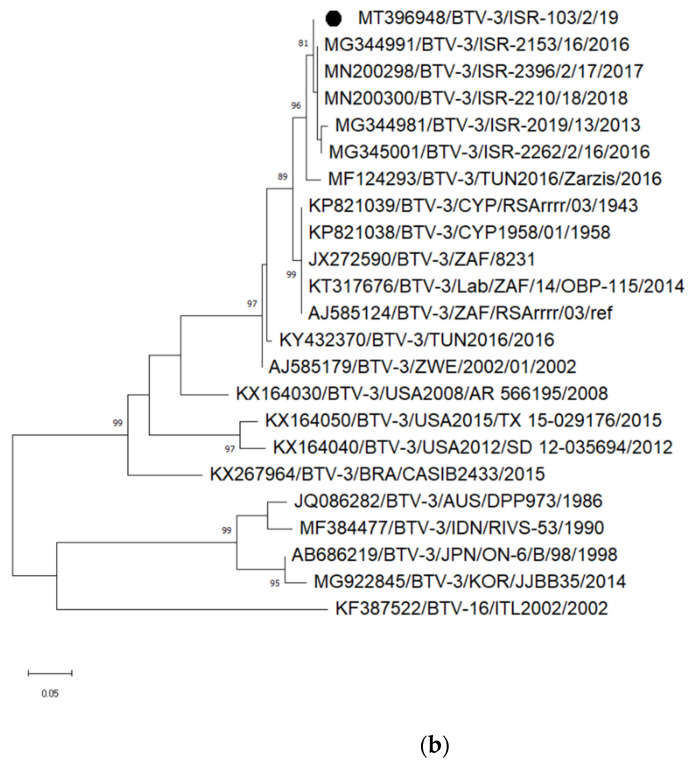

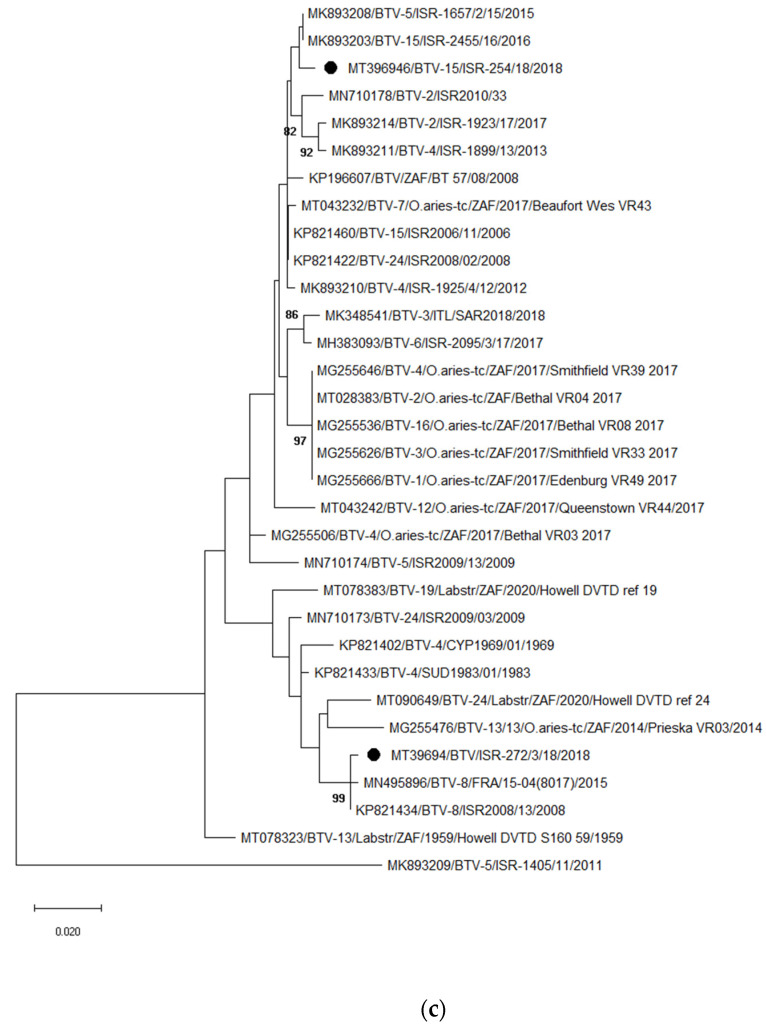
Phylogenetic analyses based on partial sequences of bluetongue viruses. (**a**) BTV-4, segment 2 (Seg-2); (**b**) BTV-3, Seg-2; (**c**) previous local (Israeli) strains and newly sequenced strains, Seg-5. The sequences obtained from NCBI GenBank are labelled by accession number/serotype/location/isolate/year (when all data is available in GenBank or the corresponding publications) and sequences generated in the present study are marked by black dots. Nucleotide sequences were analyzed using the Maximum Likelihood method and Tamura-Nei model. Statistical support for nodes was obtained by bootstrapping (1000 replicates); only values ≥ 70 % are shown. Scale bars indicate nucleotide substitutions per site.

BTV-4 was identified in 2018 by conventional PCR subsequent to a positive result of the pan-BTV RT-PCR in the brain of a newborn calf (submission number ISR-2367/18), which showed clinical signs indicative for a neurological disease. In the same year, another BTV-4 strain was found in an ovine placenta (submission number ISR-277/18). Partial sequences of segment 2 were generated for both newly detected BTVs and both showed the highest identities (>99%) to strains previously detected in Israel and some other eastern Mediterranean countries (Figure 2a). In January 2019, a different serotype, namely BTV-3, was identified in an ovine placenta from an abortion case (ISR-103/2/19). In the phylogenetic tree based on segment 2, the newly detected BTV-3 strain clustered closely together with strains detected in Israel between 2013 and 2018 (Figure 2b). In addition, BTV-9 was identified in two placentas, one lung and one brain sample of two abortion sheep cases in 2019 by a type-specific RT-qPCR [43]; sequence analyses were not performed. Another brain sample (ISR-272/3/18) collected in 2018 from a newborn lamb showing neurological clinical signs, tested weak positive in the pan-BTV RT-qPCR (Cq values of 36.33 to 38.5 during repeated testing). A fragment of 411 nucleotides of segment 5 could be amplified and the generated sequence suggested that the virus is related to Israeli BTV-8 strains. Further sequences could not be generated from this sample, most likely due to the low genome load.

Additionally, the presence of BTV-15 was confirmed in an ovine fetus and placenta of the abortion case ISR-254/18. A fragment of 412 nucleotides of segment 5 was amplified and BLAST analyses revealed the closest relationship of the strain ISR-254/18 to Israeli BTV-5 and Israeli BTV-15 strains isolated in 2015 and 2016. Segment 2 sequences could not be generated.

#### 3.3.3. Typing of Pestiviruses

**Figure 3:**
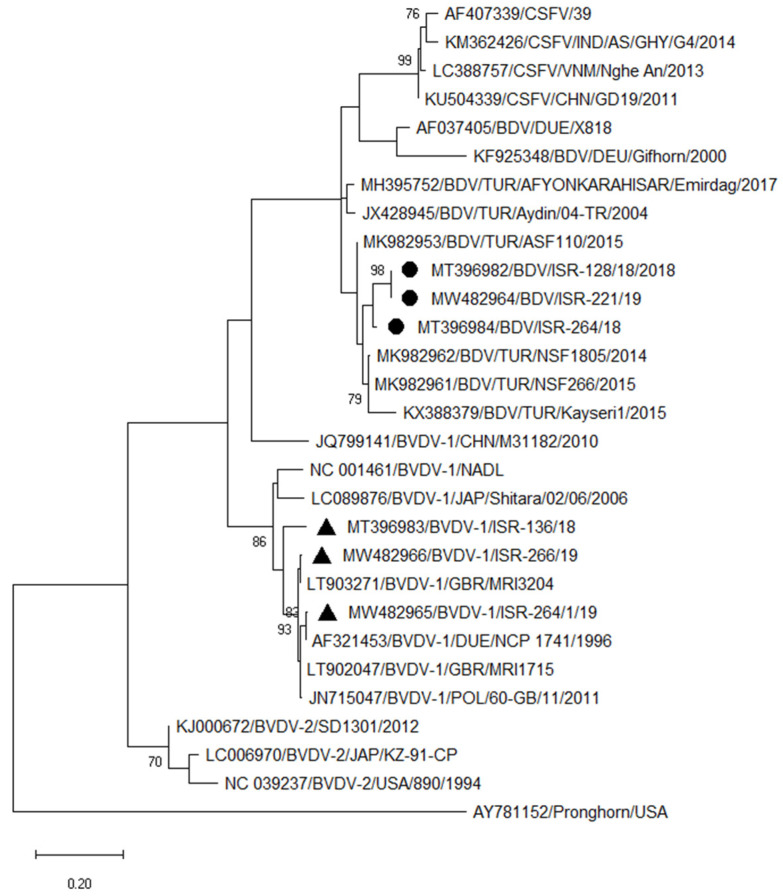
Phylogenetic analyses based on 5′UTR sequences of pestiviruses. The virus species BVDV-1 (*Pestivirus A*), BVDV-2 (*Pestivirus B*) and BDV (*Pestivirus D*) are included, pronghorn antelope pestivirus (*Pestivirus E*) is used as an outgroup. The sequences obtained from NCBI GenBank for comparison are labelled by accession number/virus species/location/isolate/year (when all data is available in GenBank or the corresponding publications). BVDV sequences generated in the present study are marked by black triangles and BDV sequences by black dots. Nucleotide sequences were analyzed using the maximum likelihood method and Kimura 2-parameter model. Statistical support for nodes was obtained by bootstrapping (1000 replicates); only values ≥ 70 % are shown. Scale bars indicate nucleotide substitutions per site.

Seven of the 13 samples obtained from 11 cases, which tested positive by the panpesti RT-qPCR, were typed. For species identification and subtyping, the 5′ UTR was sequenced. BDV was identified in a bovine fetus (ISR-264/18 strain) and an ovine fetus (ISR-128/18 strain) in 2018 and another ovine fetus (ISR-121/19 strains) in 2019. In the phylogenetic tree, these BDV strains cluster closely together (Figure 3). Further, BVDV type 1 was identified in an aborted cattle fetus in 2018 (ISR-136/18) and two bovine fetuses in 2019 (ISR-264/1/19 and ISR-266/19) (Table 1, Figure 3). Based on the 5′UTR sequence, all BVDV strains belonged to subtype BVDV-1b and are closely related to European strains.

## 4. Discussion

Embryogenic deaths, abortions, stillbirths and increasing numbers of gestation abnormalities in a herd or flock are responsible for major economic losses in livestock farming. However, potential causes of abortion are numerous and can be determined only in a subset of cases [2,3,52]. In a study from the 1990s on cattle abortion in Switzerland, the cause of the abortion was diagnosed in only 30% of the cases [53]. Though the diagnostic options and methods have markedly improved during the last decades, there is still no universal diagnostic procedure to identify and differentiate the diverse causes of abortion, which ranges from multiple pathogens to toxic agents, stress or inherited genetic abnormalities. Nevertheless, the first step in investigating increased rates of abortion or other pregnancy anomalies (e.g., fetal malformation) should be a full step-wise post-mortem examination of representative cases. In addition, diagnostic toolboxes or workflows were introduced more recently, especially for bacteria-induced miscarriages in small ruminants [54,55,56].

In this study, we focused on the detection of viruses suspected to be regularly involved in gestation abnormalities, among them members of the families *Peribunyaviridae*, *Reoviridae, Flaviviridae* and *Herpesviridae* [3]. We found that orbiviruses (BTV and EHDV), peribunyaviruses (AKAV and SHUV) and pestiviruses (BVDV and BDV) are frequently present in abortion or stillbirth cases in Israel. In 21.8 % of investigated cases (213 out of 976), a viral abortifacient could be detected. The remaining cases might be induced by one of the aforementioned other causes. An alternative explanation for a subset of cases could be that the viral infection that triggered a lesion in the fetus may have preceded the abortion by several weeks, or especially in cattle with their nine-months gestation, even by some months. By the time of abortion or birth the pathogen may no longer be detectable, or evidence may have become obliterated by autolysis. For this reason, an actual role of viral abortifacients could have been underestimated.

Of note, vector-borne pathogens such as BTV, AKAV and SHUV were often detected. When considering the data of the last two years of investigation, when tests for BTV, simbuvirus and pestivirus detections were done in parallel, BTV was found in 21 of the 267 ovine abortion cases and two of the 142 bovine cases. As BTV, simbuviruses were likewise detectable frequently, though cases numbers varied between the years. In 2016 and 2018, the total numbers of submitted samples obtained from abortion cases were higher than in the other years of the study and, in addition, AKAV was found in a higher percentage of submitted samples in 2016 and 2018 than in 2015, 2017 or 2019, suggesting virus circulation to a larger extent. However, whether this is caused by a new virus introduction or reflects the cyclic pattern of re-circulation as seen regularly for simbuviruses in endemically affected regions cannot be determined with certainty. For SBV, regular re-emergence to a larger extent is described in Central Europe, and AKAV, Aino virus and Peaton virus cause regular epizootics in Japan and AKAV in Australia [57,58,59,60,61]. Unfortunately, viral sequences from previous AKAV outbreaks in the Middle East are not available for comparison in order to demonstrate whether this pattern of virus circulation also applies for AKAV in Israel.

Besides the frequent detection of individual viruses, the situation regarding simbuviruses and orbiviruses is further complicated in Israel by the co-circulation of several members of these virus groups, namely AKAV and SHUV of the Simbu serogroup and multiple different serotypes of BTV. Both bunyaviruses and reoviruses have a segmented RNA genome and reassortment of genomic segments are described when hosts are simultaneously infected by multiple closely related viruses [16,62,63,64,65]. Here, we identified co-infections with SHUV and AKAV, and different BTV serotypes have been circulating at the same time. Hence, there are optimal conditions for the emergence of reassorted viruses. Therefore, it is recommended to regularly generate sequences of multiple genomic segments of the detected virus strains to identify potential reassortants once they emerge.

The prevention of infections with simbuviruses or BTV and, along with it, the prevention of clinical disease, abortion, fetal malformation or potential reassortment events is complicated by the transmission mode, as insect-transmitted viruses are harder to control by means other than vaccination than are pathogens directly transmitted from animal to animal. As an example, European experiences during the BTV-8 outbreak in 2006/2007 have demonstrated that vaccination can play a major role in reducing virus circulation or even in eradicating a vector-transmitted disease from a given region [66,67], while SBV, for which no vaccination campaign is in place, is endemic in Central Europe since its initial emergence about 10 years ago [61,68]. In contrast, BVDV has been successfully eradicated from several countries without vaccination after the implementation of control programs that are based on systematic control, the identification and rapid removal of infected animals, movement restrictions and further biosecurity measures [69,70]. However, in Israel, such BVDV control programs are not in place and, consequently, this virus and the related BDV could be detected in a relatively high proportion of gestations with abnormal courses. By the antigen ELISA, 12 out of the investigated 278 bovine samples tested positive, as did 30 of the 324 ovine, six of the 79 caprine samples from abortion cases, and four out of the 16 wild ruminants. By PCR, seven of 126 bovine samples and 5 of 247 ovine samples scored positive. Hence, positive results were obtained by both methods. However, a direct comparison of percentages is difficult due to the varying and sometimes low sample numbers per year and species and missing data about the test sensitivities for the study population, which could have been resolved by parallel testing of every sample by both methods. Nevertheless, in the context of ring trials using a limited number of well-characterized samples, both methods obtained equivalent results [71] and, therefore, we consider the results of both test systems valid. Furthermore, sequence analyses demonstrated that both applied methods enabled the detection of diverse strains of different pestivirus species. Besides, the detection of BDV in cattle illustrated once again the potential of trans-species transmission of certain pestivirus species.

To prevent BTV infections, in Israel inactivated vaccines against BTV-4 and BTV-8 are used mostly in sheep flocks, as they have higher economic loses from BTV compared to goat flocks and cattle herds. However, the prevention of bluetongue disease by immunization against only two serotypes is hampered by the frequent introduction of additional new serotypes, as shown by the detection of BTV-3, BTV-9 or BTV-15 in the present study.

In conclusion, it was shown that various viruses are involved in abnormal courses of pregnancy in ruminants in Israel. The current work may allow for a better understanding of the epidemiological situation of viral abortifacients in Israel, which can help in the developing of future preventive measures.

## Figures and Tables

**Table 1:** Data on detection and identification of viral genome or antigen in tissue and blood samples collected from aborted fetuses or malformed newborn animals of domestic and non-domestic ruminants. From cattle and sheep, placenta, brain, internal organs (i.organs) and blood were investigated, while for goats and other ruminant species placenta, brain and internal organs were included. The number of positive results/number of investigated samples per animal species and clinical specimen is provided.

		Cattle				Sheep				Goat			Other Ruminant Species		
Year	Pathogen	Placenta	Brain	I. Organs	Blood	Placenta	Brain	I. Organs	Blood	Placenta	Brain	I. Organs	Placenta	Brain	I. Organs
2015	BTV	-	-	1/2	-	1/1	-	1/4	-	-	-	-	-	-	-
	EHDV	6/13	7/16	0/6	-	-	-	-	-	-	-	-	-	-	-
	pestiviruses (antigen ELISA)	0/12	-	4/71	-	0/1	-	9/59	-	-	-	2/22	-	-	2/6
	simbuviruses	3/13	4/46	2/10	0/3	1/2	2/36	1/5	-	-	1/15	1/1	-	0/13	0/1
	-AKAV	1	1	0	0	0	2	0	-	-	0	0	-	-	-
	-SHUV	1	3	2	0	0	0	1	-	-	1	1	-	-	-
	-AKAV + SHUV	1	0	0	0	1	0	0	-	-	0	0	-	-	-
2016	BTV	-	-	1/10	-	-	-	0/18	-	-	-	0/5	-	-	0/2
	EHDV	0/10	0/7	0/10	-	-	-	-	-	-	-	-	-	-	-
	pestiviruses (antigen ELISA)	0/16	-	5/59	-	0/9	-	11/73	-	0/3	-	0/25	-	-	0/2
	simbuviruses	0/27	6/52	-	0/1	8/50	20/81	0/2	-	4/18	2/16	0/2	0/2	0/13	-
	-AKAV	0	1	-	0	3	9	0	-	3	2	-	-	-	-
	-SHUV	0	3	-	0	5	11	0	-	1	0	-	-	-	-
	-untyped simbuviruses	0	2	-	0	0	0	0	-	0	0	-	-	-	-
2017	BTV	-	-	0/7	-	-	-	0/8	-	-	-	-	-	-	0/3
	EHDV	0/1	0/1	0/1	-	-	-	-	-	-	-	-	-	-	-
	pestiviruses (antigen ELISA)	0/18	-	1/49	-	0/5	-	3/61	-	0/1	-	2/16	-	-	2/5
	simbuviruses	0/19	2/34	0/7	0/1	0/25	5/43	1/7	-	1/3	0/17	0/2	0/1	0/12	-
	-AKAV	0	0	0	0	0	1	0	-	0	-	-	-	-	-
	-SHUV	0	0	0	0	0	1	1	-	1	-	-	-	-	-
	-untyped simbuviruses	0	2	0	0	0	3	0	-	0	-	-	-	-	-
2018	BTV	0/6	1/42	0/5	-	6/46	10/135	0/17	-	0/3	0/11	-	-	-	0/3
	-BTV-4	0	1	0	-	1	0	-	-	0	0	-	-	-	-
	-BTV-8	0	0	0	-	0	1?	-	-	0	0	-	-	-	-
	-BTV-15	0	0	0	-	1	1	-	-	-	-	-	-	-	-
	-untyped BTV	0	0	0		4	8	0	-	-	-	-	-	-	-
	pestiviruses (antigen ELISA)	-	-	2/39	-	0/2	-	5/96	-	-	-	1/6	-	-	0/1
	pestiviruses (PCR)	-	2/35	-	-	0/46	1/131	-	-	0/3	0/11	-	0/1	0/4	-
	-BVDV-1	-	1	-	-	-	0	-	-	0	0	-	0	0	-
	-BDV	-	1	-	-	-	1	-	-	0	0	-	0	0	-
	BoHV-1	0/2	1/26	0/4	-	-	-	-	-	-	-	-	-	-	-
	BEFV	0/2	0/26	0/4	-	-	-	-	-	-	-	-	-	-	-
	simbuviruses	0/6	3/58	0/2	0/4	24/46	32/136	-	0/2	2/3	6/14	0/1	0/1	0/1	-
	-AKAV	-	0	-	-	18	23	-	0	2	6	-	-	-	-
	-SHUV	-	1	-	-	2	1	-	0	0	0	-	-	-	-
	-untyped simbuviruses	-	2	-	-	4	8	-	-	-	-	-	-	-	-
2019	BTV	1/24	0/69	0/19	-	4/62	3/70	2/19	-	0/2	1/11	0/4	-	0/4	-
	-BTV-3	0	-	-	-	1	0	0	-	0	0	-	-	0	-
	-BTV-9	0	-	-	-	2	1	1	-	0	0	-	-	0	-
	-untyped BTV	1	-	-	-	1	2	1	-	-	-	-	-	-	-
	EHDV	0/2	-	0/1	-	-	-	-	-	-	-	-	-	-	-
	pestiviruses (antigen ELISA)	-	-	0/14	-	2/2	-	0/16	-	-	-	1/6	-	-	0/2
	pestiviruses (PCR)	0/10	2/29	3/38	-	1/24	1/29	3/48	-	0/2	0/8	0/5	0/1	-	0/3
	-BDV	0	0	0	-	0	1	0	-	-	-	-	-	-	-
	-BVDV-1	0	2	0	-	0	0	0	-	-	-	-	-	-	-
	-untyped pestiviruses	0	0	3	-	1	0	3		-	-	-	-	-	-
	BoHV-1	0/19	1/58	0/15	-	-	-	-	-	-	-	-	-	-	-
	BEF	0/19	0/58	0/15	-	-	-	-	-	-	-	-	-	-	-
	simbuviruses	4/24	5/69	0/5	1/1	6/62	8/72	0/3	-	0/2	0/12	-	0/1	0/7	-
	-AKAV	1	4	0	0	3	4	-	-	-	-	-	-	-	-
	-SHUV	3	1	0	1	1	2	-	-	-	-	-	-	-	-
	-untyped simbuviruses	0	0	0	0	2	2	-	-	-	-	-	-	-	-
total	BTV	1/30	1/111	2/43	-	11/109	13/205	3/66	-	0/5	1/22	0/9	-	0/4	0/8
	-BTV-3	0	0	0	-	1	0	0	-	0	0	0	-	0	0
	-BTV-4	0	1	0	-	1	0	0	-	0	0	0	-	0	0
	-BTV-8	0	0	0	-	0	1?	0	-	0	0	0	-	0	0
	-BTV-9	0	0	0	-	2	0	0	-	0	0	0	-	0	0
	-BTV-15	0	0	0	-	1	1	-	-	-	-	-	-	-	-
	-untyped BTV	1	0	2		6	11	3	-	-	-	-	-	-	-
	EHDV	6/26	7/24	0/18	-	-	-	-	-	-	-	-	-	-	-
	BoHV-1	0/21	2/84	0/19	-	-	-	-	-	-	-	-	-	-	-
	BEFV	0/21	0/84	0/19	-	-	-	-	-	-	-	-	-	-	-
	pestiviruses (ELISA)	0/46	-	12/232	-	2/19	-	28/305	-	0/4	-	6/75	-	-	4/16
	pestiviruses (PCR)	0/10	4/64	3/38	-	1/70	2/160	3/48	-	0/5	0/19	0/5	0/2	0/4	0/3
	-BDV	0	1	0	-	0	2	0	-	-	-	-	-	-	-
	-BVDV-1	0	3	0	-	0	0	0	-	-	-	-	-	-	-
	-untyped pestiviruses	0	0	3	-	1	0	3	-	-	-	-	-	-	-
	simbuviruses	7/89	20/259	2/24	1/10	39/185	67/368	2/17	0/2	7/26	9/74	1/6	0/5	0/46	-
	-AKAV	2	6	0	0	24	39	0	0	5	8	0	-	-	-
	-SHUV	4	8	2	1	8	15	2	0	2	1	1	-	-	-
	-AKAV + SHUV	1	0	0	0	1	0	0	0	0	0	0	-	-	-
	-untyped simbuviruses	0	6	0	0	6	13	0	0	0	0	0	-	-	-

## Data Availability

The sequences generated in this study were uploaded to the INSDC under the accession numbers listed in Supplementary Table S2.

## Supplementary Materials

The following are available online at https://www.mdpi.com/article/10.3390/v13112136/s1, Table S1: List of primers used for identification and sequencing of viruses circulating in Israel. BTV–bluetongue virus, AKAV–Akabane virus, TINV–Tinaroo virus. Seg–genomic segment. Table S2: Information of viral strain IDs, sample origin, date of sampling, accession number and genome region sequenced in the current study. BTV–bluetongue virus, AKAV–Akabane virus, SHUV–Shuni virus, BDV–border disease virus, BVDV–bovine viral diarrhea virus
