# Supplementary material for: Identification and Genetic Characterization of Viral Pathogens in Ruminant Gestation Abnormalities, Israel, 2015–2019
Source: Viruses. 2021 Oct 22;13(11):2136. doi: 10.3390/v13112136 (PMC8619439; doi:10.3390/v13112136)
Supplement: Supplementary file 1 [file viruses-13-02136-s001.zip › Table S1-primers_kw.pdf]

Table S1. List of primers used for identification and sequencing of viruses circulating in Israel. BTV – bluetongue virus, AKAV – Akabane virus, TINV – Tinaroo virus, Seg – genomic segment

Virus/serotype/region	name	sequence	length of PCR fragment	source
Simbuvirues	Uni-S-59F	GAT GTW CCW CAA CGG AAT	215	[1]
	Uni-S-254R	TGG GGA AAA TGG TTA TTA AC		
BTV/Seg-5	UNI-NS1-40F	GCGCTTTTTGAGAAAATACAAC	411	This study
	UNI-NS1-428R	AGAGAATCATCCAATCTAACTCT		
BTV-4/Seg-2	VP2-S4-1F	TGTTCCCAAACTAGACATCGTTAG	493	
	VP2-4-1R	TACCCATTTCTCCGAACCTA		[2]
BTV-4/24/Seg-2	BT4-10-24-1F	ATG GAG GAR TTY GTC ATW CCW GT	329	
	BT4-10-24-330R	TCR TCR ATR GCY CAY TTC ATC CA	332	[2]
BTV/Seg-1	UNI-VP1-1F	GTTAAAATGCAATGGTCGCAAT	829	This study
	UNI-VP1-810R	CCCCACATCTTYACAAACCA		
AKAV&TINV/ M-segment	AkaTinM-1F	GTA GTG AAC TAC CAC AAC AAA ATG	659	This study
	AkaTinM-636R	RYT CTG CAT TTG TAC AGA TTG AY		
AKAV&TINV/ S-segment	AKAV-S-2F	GTA GTG AAC TCC ACT ATT AAC TAC G	845	This study
	AKAV-S-822R	CTAT AAA CAA TAA AAT CCA AGC AG		
AKAV&TINV/ L-segment	AkaTinL-1196F	TGT AAT GTG GGA ACA GCA ATT TGT	386	This study
	AkaTinL-1560R	CTG CCA RCA TGT TTT TCA TTA A		
Pestiviruses/5'-UTR	BVD1-F	GGT AGC AAC AGT GGT GAG TTC	257	[3]
	BVD2-F	AGC GGT AGC AGT GAG TTC ATT	257	
	BVD-R	CAA CTC CAT GTG CCA TGT AC		
BTV-3/Seg-2	3VP2-1F	GTT AAA AAC GCT GTC CCG AGA	658	[2]
	3VP2-637R	GAG CGC CCA CTC TAA ATT CCT C		

References

1. Golender, N.; Bumbarov, V.Y.; Erster, O.; Beer, M.; Khinich, Y.; Wernike, K. Development and validation of a universal S-segment-based real-time RT-PCR assay for the detection of Simbu serogroup viruses. J Virol Methods 2018, 261, 80-85, doi:10.1016/j.jviromet.2018.08.008.
2. Golender, N.; Bumbarov, V.; Eldar, A.; Lorusso, A.; Kenigswald, G.; Varsano, J.S.; David, D.; Schainin, S.; Dagoni, I.; Gur, I., et al. Bluetongue serotype 3 in Israel 2013-2018: Clinical manifestations of the disease and molecular characterization of Israeli strains. Frontiers in veterinary science 2020, 7, 112, doi:10.3389/fvets.2020.00112.
3. Schaarschmidt, U.; Schirrmeier, H.; Strebelow, G.; Wolf, G. Detection of border disease virus in a sheep flock in Saxonia. Berl Munch Tierarztl 2000, 113, 284-288.
