# Supplementary material for: Identification and Genetic Characterization of Viral Pathogens in Ruminant Gestation Abnormalities, Israel, 2015–2019
Source: Viruses. 2021 Oct 22;13(11):2136. doi: 10.3390/v13112136 (PMC8619439; doi:10.3390/v13112136)
Supplement: Supplementary file 1 [file viruses-13-02136-s001.zip › Table S2_kw.pdf]

Table S2. Information of viral strain IDs, sample origin, date of sampling, accession number and genome region sequenced in the current study. BTV – bluetongue virus, AKAV – Akabane virus, SHUV – Shuni virus, BDV – border disease virus, BVDV – bovine viral diarrhea virus

strain ID	acc. number	date of sampling	species	tissue	virus	segment/sequenced region
ISR-254/18	MT396946	11-Dec-18	sheep	placenta	BTV-15	Seg-5/40-450
ISR-272/3/18	MT396947	12-Dec-18	sheep	brain	BTV-8?	Seg-5/40-450
ISR-103/2/19	MT396948	03-Jan-19	sheep	placenta	BTV-3	Seg-2/1-652
ISR-277/18	MT396949	19-Dec-18	sheep	placenta	BTV-4	Seg-2/172-664
ISR-2367/18	MT396950	02-Dec-18	cattle	brain	BTV-4	Seg-1/7-787
ISR-2367/18	MT396951	02-Dec-18	cattle	brain	BTV-4	Seg-2/8-787
ISR-230/18	MT396952	21-Oct-18	sheep	placenta	AKAV	L-Seg/1194-1571
ISR-254/2/16	MT396953	11-Dec-16	goat	brain	AKAV	L-Seg/1194-1570
ISR-255/18	MT396954	26-Nov-18	sheep	placenta	AKAV	L-Seg/1194-1569
ISR-257/3/18	MT396955	04-Dec-18	sheep	placenta	AKAV	L-Seg/1194-1579
ISR-260/2/18	MT396956	06-Dec-18	sheep	placenta	AKAV	L-Seg/2643-3121
ISR-285/2/18	MT396957	31-Dec-18	sheep	placenta	AKAV	L-Seg/1194-1579
ISR-2508/2/18	MT396958	13-Dec-18	sheep	spinal cord	AKAV	L-Seg/1194-1579
ISR-146/2/16	MT396959	28-Feb-16	goat	placenta	AKAV	M-Seg/2-657
ISR-227/18	MT396961	15-Oct-18	sheep	placenta	AKAV	M-Seg/21-633
ISR-230/18	MT396962	21-Oct-18	sheep	placenta	AKAV	M-Seg/2-652
ISR-236/18	MT396963	05-Nov-18	sheep	placenta	AKAV	M-Seg/7-657
ISR-244/15	MT396964	13-Dec-15	cattle	brain	AKAV	M-Seg/2-657
ISR-244/18	MT396965	15-Nov-18	goat	brain	AKAV	M-Seg/8-655
ISR-254/2/16	MT396966	11-Dec-16	goat	brain	AKAV	M-Seg/23-655
ISR-255/18	MT396967	26-Nov-18	sheep	placenta	AKAV	M-Seg/2-655
ISR-257/3/18	MT396968	04-Dec-18	sheep	placenta	AKAV	M-Seg/2-657
ISR-260/15	MT396969	31-Dec-15	sheep	brain	AKAV	M-Seg/2-657
ISR-285/2/18	MT396970	31-Dec-18	sheep	placenta	AKAV	M-Seg/2-657
ISR-219/18	MT396972	10-Oct-18	goat	placenta	AKAV	S-Seg/20-747
ISR-230/18	MT396973	21-Oct-18	sheep	placenta	AKAV	S-Seg/34-757
ISR-236/2/18	MT396974	05-Nov-18	sheep	placenta	AKAV	S-Seg/37-801
ISR-232/1/18	MT396975	23-Oct-18	goat	placenta	AKAV	S-Seg/32-797
ISR-247/2/16	MT396976	22-Nov-16	goat	placenta	AKAV	S-Seg/1-780
ISR-254/2/16	MT396977	11-Dec-16	goat	brain	AKAV	S-Seg/1-780
ISR-255/18	MT396978	26-Nov-18	sheep	placenta	AKAV	S-Seg/40-800
ISR-257/3/18	MT396979	04-Dec-18	sheep	placenta	AKAV	S-Seg/34-798
ISR-260/2/18	MT396980	06-Dec-18	sheep	placenta	AKAV	S-Seg/31-814
ISR-285/2/18	MT396981	31-Dec-18	sheep	placenta	AKAV	S-Seg/33-770
ISR-256/16	MZ547650	11-Dec-16	sheep	placenta	AKAV	L-Seg/1-6855
ISR-256/16	MZ547651	11-Dec-16	sheep	placenta	AKAV	M-Seg/1-4297
ISR-256/16	MZ547652	11-Dec-16	sheep	placenta	AKAV	S-Seg/1-856

ISR-286/1/18	MN371396	31-Dec-18	sheep	brain	SHUV	M-Seg/3553-4317
ISR-147/19	MN371395	24-Feb-19	cattle	placenta	SHUV	M-Seg/3078-4317
ISR-170/18	MW822046	19-Jul-18	sheep	placenta	AKAV	L-Seg/full-length
ISR-170/18	MW822047	20-Jul-18	sheep	placenta	AKAV	M-Seg/full-length
ISR-170/18	MW822048	21-Jul-18	sheep	placenta	AKAV	S-Seg/full-length
ISR-128/18	MT396982	06-Mar-18	sheep	brain	BDV	'5-UTR, polyprotein/260 bp
ISR-136/18	MT396983	02-Apr-18	cattle	brain	BVDV-1	'5-UTR, polyprotein/239 bp
ISR-264/18	MT396984	06-Dec-18	cattle	brain	BDV	'5-UTR, polyprotein/258 bp
ISR-221/19	MW482964	07-Jul-19	sheep	brain	BDV	'5-UTR/247 bp
ISR-264/1/19	MW482965	28-Nov-19	cattle	lung	BVDV-1	'5-UTR/242 bp
ISR-266/19	MW482966	04-Dec-19	cattle	spleen	BVDV-1	'5-UTR/189 bp
